# Integrating *Narcissus*-derived galanthamine production into traditional upland farming systems

**DOI:** 10.1038/s41598-021-81042-9

**Published:** 2021-01-14

**Authors:** M. D. Fraser, H. E. Vallin, J. R. T. Davies, G. E. Rowlands, X. Chang

**Affiliations:** 1 Institute of Biological, Environmental and Rural Sciences, Aberystwyth University, Pwllpeiran, Cwmystwyth, Aberystwyth, SY23 4AB UK; 2 Royal Agricultural University, Stroud Rd, Cirencester, Gloucestershire, GL7 6JS UK

**Keywords:** Plant sciences, Diseases

## Abstract

Alzheimer’s disease (AD) is a disorder associated with progressive degeneration of memory and cognitive function. Galantamine is a licenced treatment for AD but supplies of the plant alkaloid that it is produced from, galanthamine, are limited. This three-year system study tested the potential to combine *Narcissus*-derived galanthamine production with grassland-based ruminant production. Replicate plots of permanent pasture were prepared with and without bulbs of *Narcissus pseudonarcissus* sown as lines into the sward. Two different fertiliser regimes were imposed. The above-ground green biomass of *N. pseudonarcissus* was harvested in early spring and the galanthamine yield determined. In the second harvest year a split-plot design was implemented with lines of *N. pseudonarcissus* cut annually and biennially. All plots were subsequently grazed by ewes and lambs and animal performance recorded. Incorporation of *N. pseudonarcissus* into grazed permanent pasture had no detrimental effects on the health or performance of the sheep which subsequently grazed the pasture. There was no consistency to the effects of fertiliser rates on galanthamine yields. There was no difference in overall galanthamine yield if *N. pseudonarcissus* was cut biennially (1.64 vs. 1.75 kg galanthamine/ha for annual combined vs biennial cuts respectively; s.e.d = 0.117 kg galanthamine/ha; ns). This study verified the feasibility of a dual cropping approach to producing plant-derived galanthamine.

## Introduction

The 2015 World Alzheimer Report on the global impact of dementia estimated that 46.8 million people worldwide were living with the condition, with this figure predicted to nearly double every 20 years, affecting 74.7 million in 2030 and 131.5 million in 2050^1^. Alzheimer’s disease (AD) is a disorder associated with progressive degeneration of memory and cognitive function that accounts for between 60 and 80% of dementia cases^2^. Galantamine is a selective, long-acting and reversible acetylcholinesterase inhibitor that has been a licenced symptomatic treatment for AD across Europe, the USA and Asia since 2000^3^. It slows down disease progression and can help to ease the symptoms of memory loss, confusion and changes in behaviour^4,5^, in turn improving quality of life and allowing patients to live independently for longer. The main source of the pharmaceutical product galantamine (galan/t/amine) has been the plant-derived alkaloid galanthamine (galan/th/amine). Galanthamine is produced by species of the Amaryllidaceae family, including *Galanthus nivalis* (common snowdrop), *Lycoris radiata* (red spider lily), *Leucojum aestivum* (summer snowflake) and *Narcissus* (daffodil) spp.^4,6^. Apart from *Narcissus* spp, these source plants are wildflowers unsuitable for agricultural production due to limitations in either resources (e.g. volume and/or cost of bulbs available) or research. Consequently, supplies of galanthamine, and thus galantamine, have been limited. Synthetic galantamine production has been explored^7,8^, but this has not proved to be an economically viable alternative at scale^6^.

A recent proof-of-principle study tested the feasibility of producing plant-derived galanthamine by integrating *N. pseudonarcissus* into upland permanent pasture and harvesting subsequent above-ground vegetative plant materials rather than bulbs^9^. It was hypothesised that cultivating the crop in marginal growing conditions could exploit relationships between exposure to stress and production of plant secondary compounds^10–13^, with biosynthesis being further activated in response to interspecies competition with forage grassland plants. The results from this trial confirmed that planting *N. pseudonarcissus* under grass competition in upland areas could offer a novel source of plant-derived galanthamine^9^. The current 3-year systems study tested an innovative dual-cropping approach based on these initial findings; increasing the scale of operation and tested the compatibility of incorporating *Narcissus* spp. into pastures subsequently grazed by sheep. Daffodils produce hundreds of different alkaloids, including some that are toxic if consumed^14^. At the same time, sheep are selective grazers and are known to avoid grazing native plant species which contain secondary compounds of a type and concentration that could compromise digestive function or health^15^. We hypothesised that the presence of alkaloids within *Narcissus* spp. would be associated with biochemical cues that would lead to avoidance of the daffodils by grazing stock. Lambs, however, are more inquisitive and naïve grazers than their dams, and may be more likely to sample unfamiliar plant species. In addition, their immature status and lower body weight may make them more susceptible to adverse effects were they to ingest *Narcissus* spp.

A secondary aspect explored within the study was the role that fertiliser addition may play in influencing daffodil and livestock performance individually and collectively. Nitrogen in particular is frequently limited in upland soils, and withdrawal of applications of this and other key nutrients leads to a significant decline in the nutritional composition of previously-improved upland swards and related stock carrying capacities^16^. Conversely, exposure to nutritional stress may increase the galanthamine concentration within daffodils^17^. The specific objectives of the current study were therefore threefold: (1) to quantify the yield of galanthamine produced by *Narcissus pseudonarcissus* when sown under upland permanent pasture; (2) to determine the impact of growing daffodils within pasture on the health and performance of grazing sheep; and (3) to investigate trade-offs between inorganic fertiliser applications and the biomass and alkaloid yields of daffodils sown into grazed permanent pasture.

## Results

Three experimental systems were imposed: (1) permanent pasture with fertiliser applied at a rate of 50 N kg/ha, 25 P kg/ha and 25 K kg/ha (rates typical of commercial practice for upland farms) (**Control**); (2) permanent pasture under-sown with *N. pseudonarcissus*, with fertiliser applied at a rate of 50 N kg/ha, 25 P kg/ha and 25 K kg/ha (**Daff/full**); and (3) permanent pasture under sown with *N. pseudonarcissus*, with fertiliser applied at a rate of 25 N kg/ha, 12.5 P kg/ha and 12.5 K kg/ha (i.e. 50% of the typical rate) (**Daff/half**).

### Year-to-year variation in growth of *N. pseudonarcissus* and impacts of management

**Figure 1:**
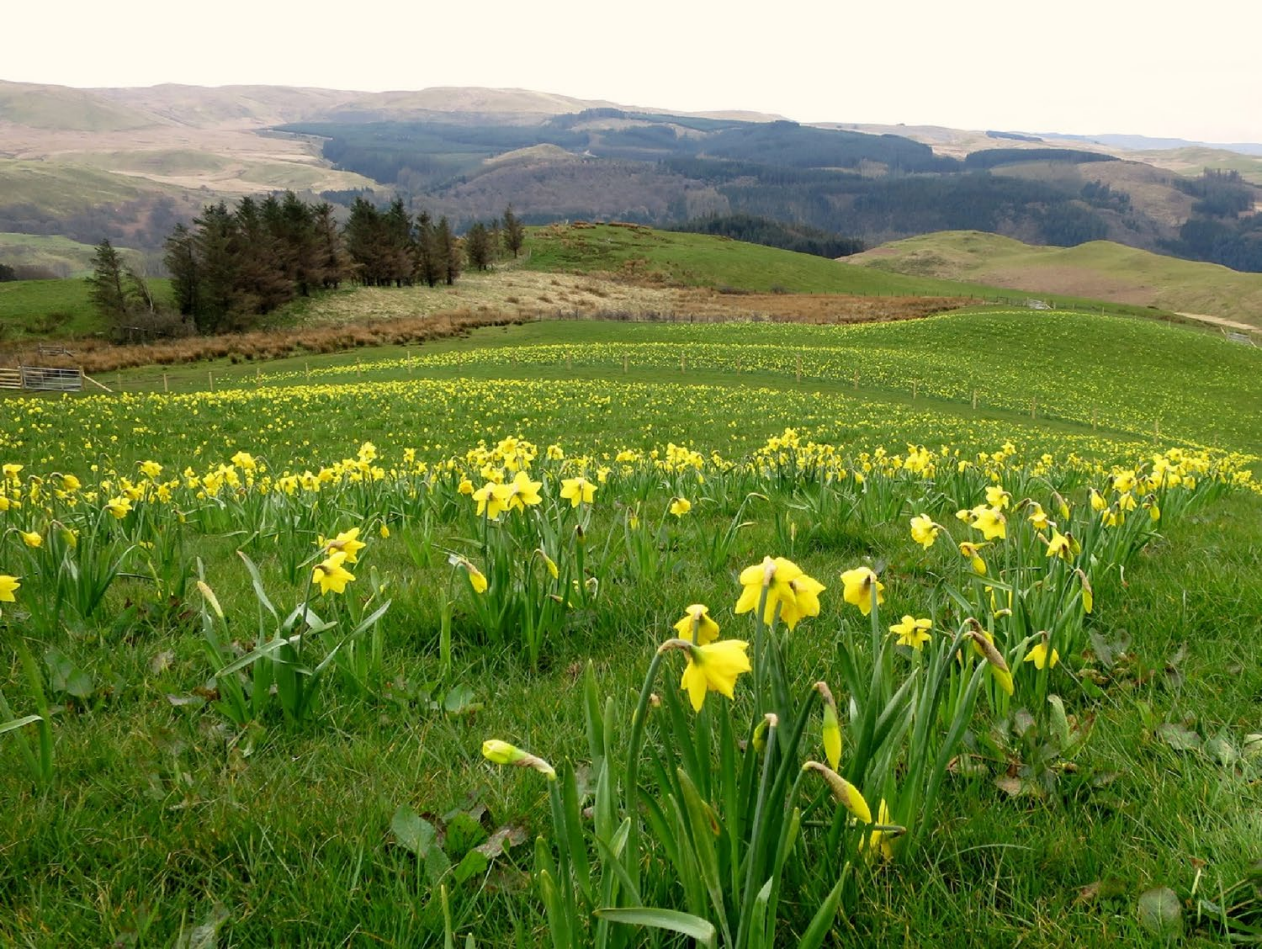
View across experimental site showing *Narcissus pseudonarcissus* cv Carlton established under improved permanent pasture at 380 m a.s.l. at a sowing rate of 4 t/ha (Photo—M. Fraser).

**Figure 2:**
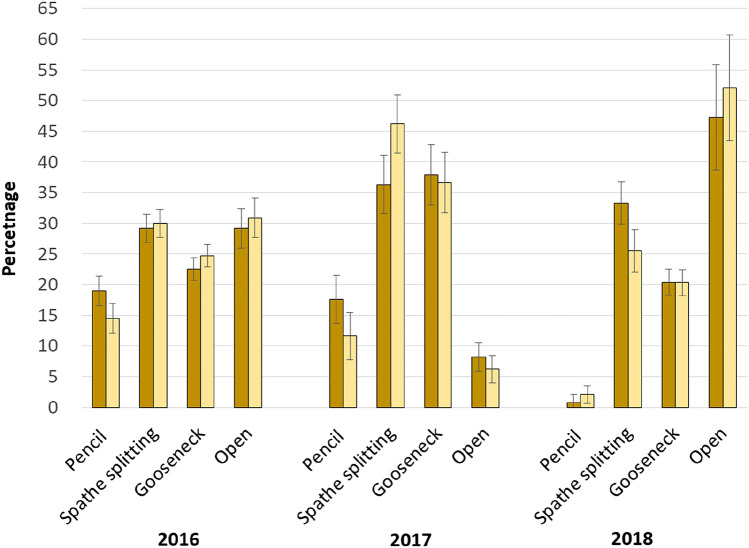
Percentage of sampled flowers of *Narcissus pseudonarcissus* cv Carlton accounted for by different growth stages at point of harvest (where dark yellow = compound fertiliser applied at a rate of 50 kgN/ha, 25 kgP/ha and 25 kgK/ha; light yellow = compound fertiliser applied at a rate of 25 kg N/ha, 12.5 kg P/ha and 12.5 kg K/ha; error bars = s.e.m.).

*Narcissus pseudonarcissus* established successfully under permanent pasture (Fig. 1). The target for harvesting the above-ground biomass in spring was when most flowers were at the gooseneck growth stage. This was a challenge to achieve due to variation in growth of individual plants, and flowers at all four growth stages were recorded within the samples taken at harvest (Fig. 2). The high percentage of flowers at the open growth stage in spring 2018 reflects the exceptionally wet spring that year, which led to the ground being unsuitable for agricultural vehicles at the optimum time for harvest. The total number of flowers recorded within the samples taken in 2016 was 111, with this declining to 40 in 2017, when there were significantly more flowers in samples from Daff/Full compared to the Daff/Half plots (19 vs. 11 flowers per 5 m; s.e.d. = 1.5 flowers; *P* < 0.001). This pattern was repeated in 2018 (38 vs. 33 flowers per 5 m; s.e.d. = 0.53 flowers; *P* < 0.05). In addition, lines which had not been harvested the previous year had more flowers (48 vs. 22 flowers per 5 m; s.e.d. = 3.3 flowers; *P* < 0.01).

In the first harvest year (2016) there was no effect of fertiliser application rate on the biomass yield of *N. psedonarcissus,* and galanthamine concentrations were also similar for the two fertiliser treatments (Table 1). In 2017, however, the biomass yield and galanthamine concentration of *N. psedonarcissus* were higher for the Daff/Full treament, leading to a markedly higher overall galanthamine yield.

**Table 1:** Estimated biomass yields of *Narcissus pseudonarcissus* (cv. Carlton) plus corresponding galanthamine yields during the first (2016) and second (2017) harvest years (where Daff/full = compound fertiliser applied at a rate of 50 kgN/ha, 25 kgP/ha and 25 kgK/ha; Daff/half = compound fertiliser applied at a rate of 25 kg N/ha, 12.5 kg P/ha and 12.5 kg K/ha).

Year		Daff/full	Daff/half	s.e.d	*F* prob
2016	FM yield (kg/ha)	1448	1765	176.8	ns
	Galanthamine conc (% FM)	0.042	0.042	0.0024	ns
	Galanthamine yield (g/ha)	594	753	91.1	ns
2017	FW yield (kg/ha)	1664	1361	107.7	< 0.01
	Galanthamine conc (% FM)	0.050	0.044	0.0024	< 0.05
	Galanthamine yield (g/ha)	831	592	62.0	< 0.001

**Figure 3:**
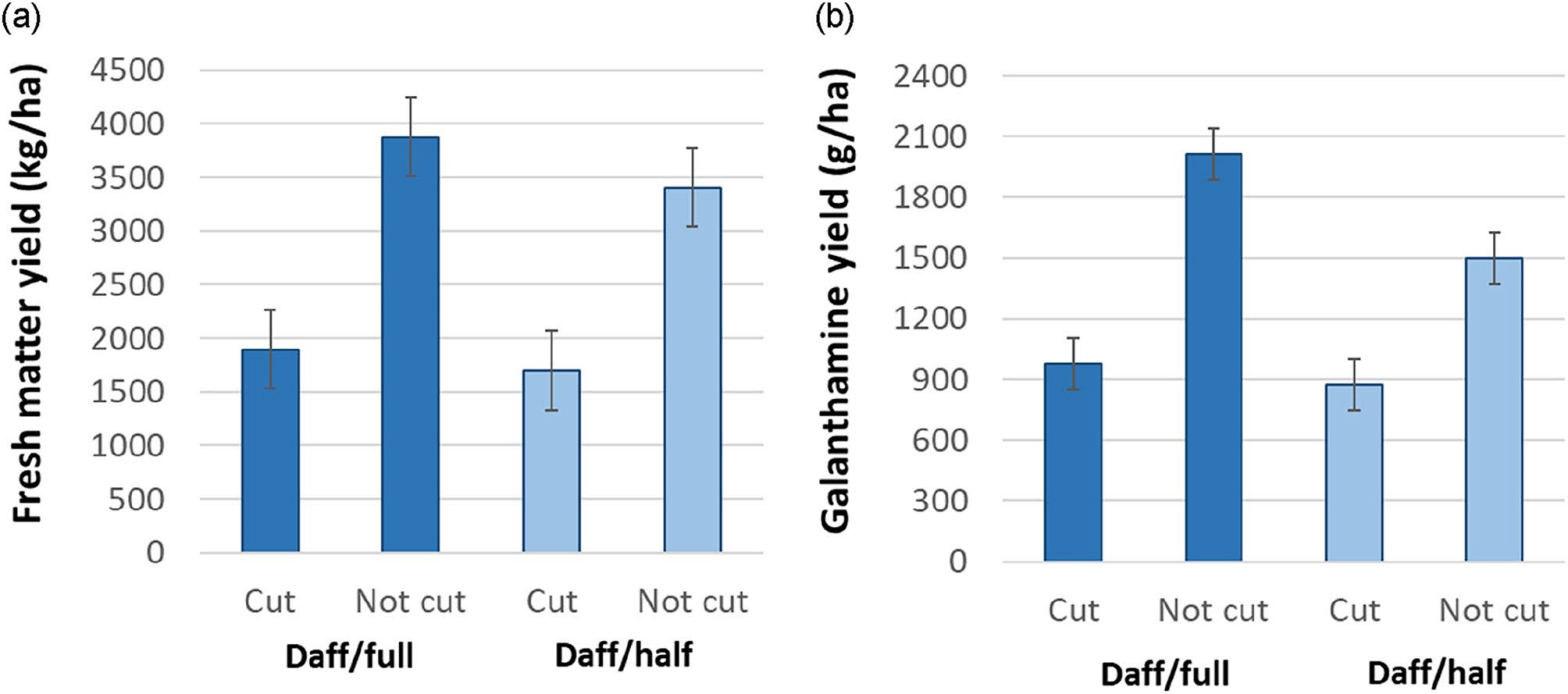
Effect of fertiliser application and cutting history on (**a**) estimated biomass yields of *Narcissus pseudonarcissus* cv Carlton, and (**b**) corresponding galanthamine yields in 2018 (where cut = harvested the previous year; not cut = no harvest the previous year; Daff/full = compound fertiliser applied at a rate of 50 kgN/ha, 25 kgP/ha and 25 kgK/ha; Daff/half = compound fertiliser applied at a rate of 25 kg N/ha, 12.5 kg P/ha and 12.5 kg K/ha; error bars = s.e.d.).

Plants which had been rested from harvesting in 2017 (second harvest year) had substantially higher biomass yields in 2018 (third harvest year) than those which had been cut the previous year (3643 vs. 1801 kg FM/ha; s.e.d. = 169.2 kg FM/ha; *P* < 0.001), whereas rate of fertiliser application had no effect (2888 vs. 2557 kg FM/ha for Daff/Full vs. Daff/Half; s.e.d. = 328.7 kg FM/ha; ns) (Fig. 3a). Neither harvesting the previous year nor fertiliser rate of application had an effect on the concentration of galanthamine (grand mean 0.051%; s.e.d. 0.0038%), which led to overall galanthamine yield reflecting biomass yield (1753 vs. 926 g/ha; s.e.d. = 64.4 g/ha; for uncut vs. cut in 2017; *P* < 0.001) (Fig. 3b). When the yields for rows harvested consecutively in 2017 and 2018 were combined and compared with the 2018 yield figures for rows left uncut in 2017 (i.e. combined annual vs biennial totals) there was no difference in the total galanthamine yield (1.64 vs 1.75 kg galanthamine/ha for annual combined vs. biennial cuts respectively; s.e.d = 0.117 kg galanthamine/ha; ns).

### Effect of incorporating *N. psedonarcissus* into permanent pasture on sheep performance

There was no effect of the incorporation of *N. psedonarcissus* into grazed permanent pasture on the liveweight gains of lambs in either 2016 or 2018, and in in 2017 performance was higher on plots where *N. psedonarcissus* had been planted than on the Control plots Table 2).

**Table 2:** Effect on liveweight gain of upland lambs prior to weaning of (1) under-sowing permanent grassland with *Narcissus pseudonarcissus* (N. pseudo)*,* and (2) reducing application rates of inorganic fertiliser (where Control = permanent pasture with fertiliser applied at a rate of 50 N kg/ha, 25 P kg/ha and 25 K kg/ha; Daff/full = permanent pasture under-sown with *N. pseudonarcissus*, with fertiliser applied at a rate of 50 N kg/ha, 25 P kg/ha and 25 K kg/ha; and Daff/half = permanent pasture under sown with *N. pseudonarcissus*, with fertiliser applied at a rate of 25 N kg/ha, 12.5 P kg/ha and 12.5 K kg/ha (i.e. 50% of the standard rate).

Year	Control	Daff/full	Daff/half	s.e.d	*F* prob		
					Treatment effect	*N. pseudo* vs. none	100% versus 50% fertiliser
2016	173	170	178	13.3	ns	ns	ns
2017	152	173	172	8.9	< 0.05	< 0.01	ns
2018	212	223	218	10.6	ns	ns	ns

### Effects of reducing fertiliser inputs on pasture and livestock performance

**Figure 4:**
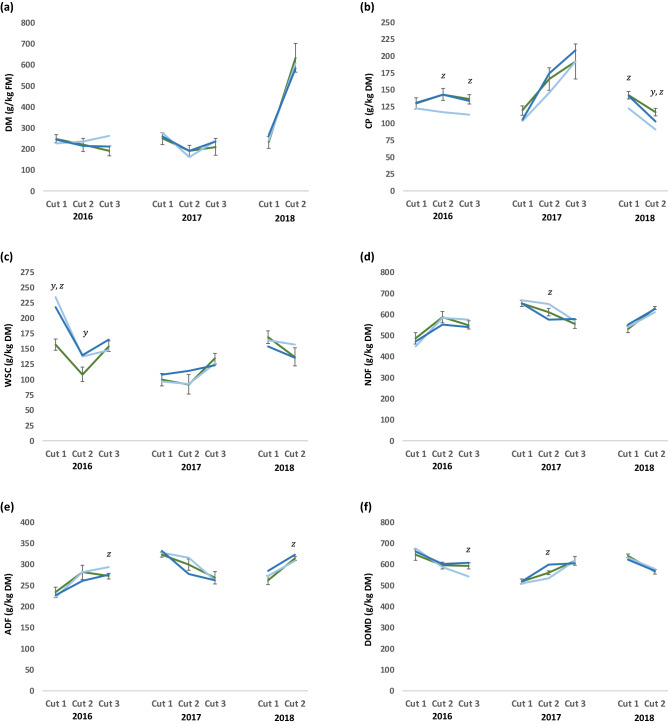
Illustration of changes in sward nutritional parameters across seasons and years, (where green line = permanent pasture with fertiliser applied at a rate of 50 N kg/ha, 25 P kg/ha and 25 K kg/ha; dark blue line = permanent pasture under-sown with *Narcissus pseudonarcissus*, with fertiliser applied at a rate of 50 N kg/ha, 25 P kg/ha and 25 K kg/ha; light blue line = permanent pasture under sown with *N. pseudonarcissus*, with fertiliser applied at a rate of 25 N kg/ha, 12.5 P kg/ha and 12.5 K kg/ha; y = significant treatment effect of sowing *N. pseudonarcissus* (*P* < 0.05); z = significant treatment effect of rate of fertiliser application (*P* < 0.05); error bars = standard errors of the difference).

Limitations in either pasture quantity or quality can influence the performance of grazing stock, and both were monitored to characterise their role in any treatment effects observed. There with no consistent effect of the presence of *N. pseudonarcissus* or fertiliser rate on sward biomass, with the figures recorded across plots and between cuts highly variable (herbage yield = 1450 kg DM/ha, 3727 kg DM/ha and 1929 kg DM/ha for Cut 1, Cut 2 and Cut 3 respectively in 2016 (s.e.d. = 296.4 kg DM/ha; *P* < 0.001); 3037 kg DM/ha, 3469 kg DM/ha and 1946 kg DM/ha for Cut 1, Cut 2 and Cut 3 respectively in 2017 (s.e.d. = 182.8 kg DM/ha; *P* < 0.001); and 5033 kg DM/ha and 2588 kg DM/ha for Cut 1 and Cut 2 respectively in 2018 (s.e.d. = 238.9 kg DM/ha; *P* < 0.001). The impact of the drought conditions in 2018 are evident in the DM content of the samples from the second (and last) cut that summer compared to the previous years (Fig. 4). Overall, the planting of *N. pseudonarcissus* into the pasture had little impact on the nutritional value of the grassland. The main exception was higher concentrations in the WSC concentrations of plots incorporating *N. pseudonarcissus* during the initial cuts in the first year. Reducing the quantity of N, P and K applied as fertiliser had a greater impact and led to increases in sward fibre concentrations and associated reductions in digestibility (Fig. 4). Concentrations of CP were also sensitive to fertiliser application rates. However, despite these differences in pasture nutritional value, rate of fertiliser application did not significantly affect lamb growth (Table 2). This may be due to ewes mobilising body stores to compensate for shortcomings in nutrient supply.

## Discussion

Upland areas within the UK and northern Europe are characterised by poor growing conditions that are largely brought about by abiotic factors such as low temperatures, exposure, and a shortage of major soil nutrients. These challenges generally limit agricultural production in these less favoured areas to grassland-based livestock systems, and for decades such farming systems have been heavily reliant upon subsidy support to be economically viable. Planting *N. psedonarcissus* into existing grassland could offer a triple win scenario whereby (1) traditional upland farming systems, and related rural populations, are maintained and have their economic viability increased, (2) AD patients have improved access to a proven treatment, and (3) the environmental impacts of galanthamine production are reduced (since wildflowers are not being picked and land is not being ploughed).

The concentration of galanthamine in *N. pseudonarcissus* has been shown to vary between different varieties^18^. The variety Carlton used in this study is considered to have potential as a commercial source of galantamine as a result of producing relatively high concentrations of galanthamine in the bulbs and there being good availability of large volumes of planting stock^19^. Galanthamine concentrations in leaves from *N. pseudonarcissus* have been found to be steady until the plant fully flowers, and then decrease^20^. Thus, by cutting at the gooseneck growth stage the biomass yield should be maximised at higher concentrations of galanthamine. During the experiment there was considerable between-plant variation in growth stage at harvest despite the bulbs coming from a single nursery, being the same size, and being planted at the same time. One factor that may have contributed to this variability was the orientation of bulbs at planting since they were dropped into the planting groove at random. In a small-scale trial to test the theory that bulb orientation would influence time to emergence replicated rows of bulbs by hand with the apex either pointing up, down or sideways. There was a delay of nearly a week in shoots emerging from those bulbs planted sideways compared to those sown pointing up, and a further week’s delay in the emergence of those pointing downwards relative to those planted sideways (*M Fraser, unpublished data*).

Yields of green material were in keeping with the average from the previous pilot study when bulbs were planted 5 cm apart^9^. Given that the height for the experimental line cuts in both cases (approximately 3 cm) was lower than would be expected by a commercial harvester (> 10 cm), plus field losses were minimal due to manual collection of material, the values recorded will overestimate those achievable at scale. The concentrations of galanthamine were higher than those recorded during a study harvesting bulbs rather than above-ground material^17^, higher than concentrations previously reported for above-ground *N. pseudonarcissus* biomass^21^, and similar to those from the earlier proof-of-principle study^9^. While these findings would support the theory that the imposition of greater plant-plant competition when growing in grassland elicits a greater stress response leading to greater galanthamine production, further research is required to verify and explore such a relationship.

There may be a variety of reasons why *N. pseudonarcissus* produce alkaloids such as galanthamine, including protection against grazing animals, as well as fungi, insects and bacteria^22^. Were the incorporation of daffodils into permanent pasture to compromise the health and welfare of grazing stock, a dual-cropping system would not be viable. Camera traps set up at strategic points within the plots recorded some nibbling of daffodils, but damage to the leaves was minimal (*M Fraser, pers. obs.*). Animals can develop a learned aversion to certain foodstuffs through associating the consequences of eating the plant with consumption of the plant. Eating *N. pseudonarcissus* has been shown to cause dogs to experience nausea and emesis^14^ and it is possible that the sheep also experienced such symptoms. However, there were no noticeable effects on ewe or sheep health recorded across this three-year study. Logged interventions were related to common conditions such as bacterial infections of hooves and were required with similar frequency across the different treatments. With different breeds of sheep and different individuals used in different years there can be confidence in the finding that growing of *N. pseudonarcissus* as a source of galanthamine is compatible with grassland-based sheep production when there is sufficient grass biomass available to be preferentially consumed. Were rows to be sown substantially closer than during the current study, to increase the biomass yield of *N. pseudonarcissus*, it is possible that stock would inadvertently consume more of these plants and thus experience ill-effects. Alternatively, avoidance of *N. pseudonarcissus* could lead to a ‘zone of rejection’ in which grass is also avoided. This would in effect lower pasture availability per head, which may reduce performance. The presence of *N. pseudonarcissus* plants and avoidance of the sward immediately adjacent to these will increase sward structural heterogeneity, however, which is known to have benefits for invertebrates. Unfortunately, the flowers of *N. pseudonarcissus* are of comparatively limited appeal to pollinators^23^. As indicated previously, extremes in growing conditions were experienced across the three growing seasons included in the study. While the summer of 2016 was comparatively representative of the long-term average, June 2017 was exceptionally wet, and the regional rainfall amount was 175% of the 1981–2010 average^24^. These conditions likely account for the significantly lower growth rates of the lambs on the Control that year since a large area within one of the replicates of this treatment became water-logged.

The timing of turnout of sheep onto the pastures was in keeping with historical usage of the area. Lambing would generally occur in April and ewes and new lambs would be kept on the lowest, most sheltered fields close to the farmstead for the first few weeks. Movement up to higher ground would occur from May, once the sward had responded to the application of nitrogen fertiliser. During the current study there was no effect on galanthamine concentrations or overall galanthamine yield of reducing fertiliser inputs. The findings therefore suggest there is no benefit in compromising inputs to grassland. The results also indicate that overall galanthamine yields are similar under a biennial rather than an annual harvesting regime. Reducing the frequency of harvest should be beneficial in lowering the overall cost of production. Such a management strategy may also increase bulb longevity, and this warrants further investigation. Soil compaction and pasture damage were further causes for concern given the narrowness of the harvesting window at the optimum growth stage and the likelihood of wet conditions for field operations. For this reason a harvester was chosen which could be pulled by a smaller (55 kW) tractor^25^. There were few marks left on the field surface indicating that any impacts were minimal and would be short lived.

While this study has focussed on production of galanthamine, more than 300 alkaloids have been isolated from the *Narcissus* genus, and most exhibit some form of biological activity^26^. Lycorine has been reported as having antibacterial, antiviral, and anti-inflammatory effects. It has also displayed various inhibitory properties towards multiple cancer cell lines that include, lymphoma, carcinoma, multiple myeloma, melanoma, leukemia, human A549 non-small-cell lung cancer, human OE21 esophageal cancer and more^27–29^. Haemanthamine and pancratistatine have also been studied as a novel anticancer agents due to their ability to overcome cancer cell resistance to apoptosis^30–32^, while narciclasine also has both anti-tumour and anti-inflammatory properties^33^. There is every expectation that the innovative dual land-use approach for obtaining plant-derived galanthamine outlined above could be adapted to produce alternative, or indeed multiple, pharmacological products such as these.

This study has verified the feasibility of a dual cropping approach to producing plant-derived galanthamine whereby *N. pseudonarcissus* is grown under upland permanent pasture which is subsequently grazed by livestock. The introduction of *N. pseudonarcissus* had no detrimental effects on sheep health or performance despite the presence of toxic alkaloids within the plant, indicating aversion during grazing. Altering the rate of inorganic fertiliser applied had comparatively little impact on outputs from the system. There is no loss of overall yield if a biennial cutting regime is implemented on *N. pseudonarcissus* and reducing the harvesting frequency would have benefits in terms of reduced operator costs. This dual-cropping approach could be adopted to produce other high value chemicals from *Narcissus* spp.

## Methods

### Experimental design and plot management

The experiment was conducted at the Pwllpeiran Upland Research Centre, Wales, UK across the summers of 2016, 2017 and 2018. The site was located on average 380 m above sea level in an area that receives around 1700 mm of rainfall per year. The pasture at the site had been last re-seeded in the 1990s and had been primarily sheep grazed.

Triplicate 0.9 ha plots of each planting treatment were prepared as 3 randomised blocks. The *N. pseudonarcissus* bulbs (cv. Carlton) were sown in September 2015 in lines approximately 90 cm apart at a rate of 4 t/ha using a tractor-mounted customised planter^25^. Daffodil harvesting was carried out using a modified flail harvester^25^ when the majority of flowers reached the gooseneck growth stage (i.e. were bent downwards to approximately a 45° angle but were unopened). Corresponding cutting dates were 15–19 April 2016, 3–7 April 2017 and 30 April–1 May 2018. Pre-harvest cuts (see below) in 2017 showed that the biomass yield was comparatively poor for a second harvest year. As a result, an additional split-plot harvesting treatment was imposed with half of each plot undergoing commercial harvest in 2017 and half left uncut. Alternative blocks of four lines of *N. pseudonarcissus* were cut or not within each plot. Compound fertiliser was applied after harvest at specified treatments rates across all plots using a tractor-mounted pendulum spreader.

Turnout of the sheep onto the plots occurred in May, when there was sufficient biomass to sustain the stock (sward surface heights approximately 7–8 cm). Within each year, all the plots were grazed by the same breed of upland sheep (year one = Beulah Speckle Faced ewes plus cross-bred lambs; years 2 and 3 = Llyn-type ewes and cross-bred lambs). Initial stocking was at a rate of 9 ewes and 13 lambs per hectare, with each plot carrying a similar combination of single and twin lambs. The animals chosen were selected from the pool of animals available based on body weight and body condition score and were allocated to treatments at random. Following weaning of the lambs in mid-July only the ewes were returned to the plots, in keeping with standard practice on upland farms. In 2016 and 2017 the sheep were removed in September when the sward surface height dropped to less than 5 cm and there was insufficient biomass to sustain them. In 2018 removal occurred in August as drought conditions had halted grass growth.

### Animal measurements

The work described was conducted in accordance with the requirements of the UK Animals (Scientific Procedures) Act 1986 and with the approval of the IBERS Animal Welfare and Ethical Review Board. The lambs were weighed every two weeks during the pre-weaning period plus all health and veterinary interventions required by ewes or lambs carefully noted.

### Galanthamine yields

Biomass and alkaloid yields of *N. pseudonarcissus* were determined immediately prior to simulated commercial harvest. *Narcissus pseudonarcissus* growth along a 5 m length was harvested to a height of 3 cm using electric shears (Makita DUM168Z; Makita (UK) Ltd, Milton Keynes, UK) at 12 random sites across each plot. The material from each line cut was subsequently weighed to determine fresh matter (FM) yield. All flowerstems within the samples were counted and assigned to growth stage categories (pencil; spathe splitting; gooseneck and open), before being returned to the bag. A sub-sample of the bulked material was stored at approx. − 20 °C for subsequent analysis. To determine galanthanime concentration, sections of approximately 100 mg FM were extracted in methanol and then analysed by high-performance liquid chromatography as described in Fraser et al.^9^.

### Pasture measurements

Grass quantity and quality was monitored by cutting herbage along both sides of a 1 m rule to a height of 1 cm using electric shears (Makita DUM168Z) every 4 weeks. Material was cut at 6 random sites across each plot. After cutting the fresh weight of each sample was recorded, cuts from a plot were bulked and thoroughly mixed, and two representative sub-samples were taken. The first was oven dried to constant weight at 60 °C to determine the dry matter (DM) content of the sample. The second sub-sample was frozen at approx. − 20 °C prior to being freeze-dried and milled through a 1 mm sieve in preparation for routine chemical analysis to determine total nitrogen (TN) (expressed as crude protein (CP) (TN × 6.25), water-soluble carbohydrate (WSC), neutral-detergent fibre (NDF) and acid-detergent fibre (ADF) concentrations as described in Fraser et al.^34^. Digestibility of organic matter in the DM (DOMD) was determined according to the two-stage method of Tilley and Terry^35^, adapted for the ANKOM DAISYII 220 incubator system (ANKOM Technology Corporation, Fairport, NY, USA).

### Data analysis

One-way analysis of variance (ANOVA) with field blocks as a blocking structure was used to test for overall treatment effects on lamb liveweight gain (Genstat (18th Edition); VSN International Ltd, Hemel Hempstead, UK). Orthogonal contrasts were then used to examine effects of control vs daffodil treatments, and standard vs half fertiliser rate treatments. One-way ANOVA with field block as a blocking structure was used to test treatment effects on most plant-based variables. For the analysis of the combined effects of fertiliser rate and whether harvested the previous year a split-plot ANOVA was used.
